# The Royal British Nurses' Association.—II

**Published:** 1916-07-01

**Authors:** 


### THE MATRONS' AND SISTERS' DEPARTMENT.

### THE ROYAL BRITISH NURSES' ASSOCIATION.—II.

FROM time to time, with varying success, this Corporation has instituted schemes for benefiting nurses. One of the first of these was a fund, now known as the Helena Benevolent Fund, for the relief of members in times of sickness or adversity. Another scheme, which has proved of great help to certain members of the Corporation, was that which resulted in the establishment of the Settlement Home for Nurses who have retired from active work and whose incomes are limited. Every available room in the Home is occupied, the nurses paying the nominal rent of 4s. 4d. annually. Every nurse contributes the sum of one penny weekly to a medical fund, and, on the strength of this, the Association undertakes to defray all expenses for medical attendance.

Some of the existing societies (established on co-operative principles for private nurses, owe their origin to the Royal British Nurses' Association. The first of these was the Registered Nurses' Society. Later the Society of Chartered Nurses was founded to give employment to members of the Corporation. This Society has proved a most successful one, and it still employs only registered members of the R.B.N.A. Both of these societies were founded on lines administratively separate from the Association, although owing their establishment to it. Still later the Auxiliary Nurses' Society was founded, with the laudable object of finding employment for older members, but this did not prove the success which its promoters had hoped, and ultimately it was found expedient to discontinue it. Some fifteen months ago, in order still further to help its members to find employment, the Association started another society, known as the Royal British Nurses' Co-operative Society, and so far, we are informed, the progress of this has proved most encouraging. Members of these societies receive their own earnings, less 7½ per cent. for working expenses. There is a good medical library for the use of working members, and the Recluse Club was founded some years ago to supply retired nurses with a weekly magazine.

There is a thriving branch of the Association in South Australia, with a large Home attached to it, where members may reside when temporarily out of employment. This branch Association also has a successful private staff. Many years ago an attempt was made to found a branch in South Africa, but this had to be abandoned as the nurses there are very scattered, and there were fewer resident in the Colony than is now the case.

The Corporation has representation on the Central Midwives Board. For some years it has been among those societies interested in promoting a Bill for the State Registration of Trained Nurses. At the time when the Association decided upon this course it met with considerable opposition from numbers of its own members, but a large majority declared themselves in favour of State registration, and the Corporation proceeded to draw up its Bill, which was one of those before Parliament for several years. Later, it united with other societies to form the Central Committee for the State Registration of Trained Nurses, in order to agree upon a common Bill. If all nursing authorities have not found themselves in accord with the Association's attitude towards State registration it is at least admitted that it has always shown itself a courteous opponent in controversy, and has abstained from the attitude of "I am of Paul" or "I of Apollos," which, on the part of other promoters of the measure, has effectually hindered its progress.

Altogether the Royal British Nurses' Association has played a useful part in the evolution of nursing; what may be its further history in the light of the new movement the future will reveal.

*(Concluded from page 281.)*

